# A long non-coding RNA *Leat1* mediates the hormone responsiveness of *EfnB2* during male urogenital development

**DOI:** 10.21203/rs.3.rs-3098271/v1

**Published:** 2023-07-03

**Authors:** Deidre Mattiske, Pascal Bernard, Paul E. Gradie, Richard R. Behringer, Paul A. Overbeek, Rachel J O’Neill, Tiffany Phillips, Gerard Tarulli, Andrew J. Pask

**Affiliations:** 1 School of BioSciences, The University of Melbourne, Victoria. 3010. Australia.; 2 Department of Genetics, University of Texas M.D. Anderson Cancer Center, Houston, 77030. TX, USA.; 3 Baylor College of Medicine. Houston, 77030. TX, USA; 4 Department of Molecular and Cell Biology and Institute for System Genomics. The University of Connecticut, Storrs, 06259 CT, USA.

**Keywords:** Ephrinb2, lncRNA, urethra, urogenital

## Abstract

The novel long non-coding RNA (lncRNA) *Leat1* is extraordinarily conserved in both its location (syntenic with *EfnB2,* an essential gene in anogenital patterning) and sequence. Here we show that *Leat1* is upregulated following the testosterone surge from the developing testis and directly interacts with *EfnB2,* positively regulating its expression. *Leat1* expression is suppressed by estrogen, which in turn suppresses the expression of *EfnB2*. Moreover, the loss of *Leat1* leads to reduced *EfnB2,* resulting in a severe hypospadias phenotype. The human *LEAT1* gene is also co-expressed with *EFNB2* in the developing human penis suggesting a conserved function for this gene in urethral closure. Together our data identify *Leat1* as a novel molecular regulator of urethral closure and implicate it as a target of endocrine disruption in the etiology of hypospadias.

## Introduction

Hypospadias is an abnormality of penis development that results in the misplacement of the urethral opening on the underside of the penis. It is one of the most common birth defects in the USA, affecting approximately 1 in every 125 live male births ^1^. Hypospadias varies in severity, depending on the location of the urethral opening ^2^. Distal hypospadias, where the urethra opens just below its normal location, is the most mild and common form of the disease accounting for up to 65% of all cases, while the more severe forms, where the urethra opens along the shaft, at the base of the phallus or scrotum, are less common accounting for around 35% of cases ^3^. Hypospadias is frequently associated with other abnormalities, including abdominal testes (10%) and inguinal hernias (15%) suggesting a common pathology for these male reproductive disorders ^4,5^.

Of major concern is the unexplained doubling in the incidence of hypospadias in developed countries in recent decades. This is not due to increased reporting and is too rapid to be accounted for by genetic mutations ^1,6^. Urethra internalization is tightly regulated by hormones and dependent on tightly balanced androgen and estrogen signaling in males ^7,8^. The process of urethra internalization can be blocked by exogenous estrogen, causing hypospadias in both mice and humans ^9,10^. Recent studies have also demonstrated a direct impact of increased estrogenic endocrine disrupting chemicals (EDCs) in human fetal blood on the incidence of hypospadias in infants ^11^. Despite this direct link, the hormonal targets that control penis development and urethra internalization are not well understood.

The development of the male penile urethra begins early in development, shortly after the septation of the cloaca into the hindgut and urinary tracts ^12,13^. In mice, the division of the cloaca and formation of the genital tubercle (GT) occurs between embryonic (E) days 10.5 and 13.5 ^12^. At this stage, the urethra terminates at the base of the developing GT ^12^. Urethra internalization proceeds from E14.5–17.5 ^14^ until the urethra is internalized along the entire length of the penis, terminating at the distal tip. Development of the GT and urethral closure invoves both androgen and estrogen signaling, along with several genetic pathways, including Hedgehog, fibroblast growth factor, bone morphogenetic protein and Wnt signaling ^15^. However, little is known about the interactions between EDCs and these gene networks.

Urethral closure is regulated in part by the Eph/Ephrin, bidirectional signaling molecules that mediate many patterning pathways in early embryonic development ^16,17^. Eph receptors are transmembrane receptor tyrosine kinases that, when bound to Ephrin ligands, signal forward in the Eph-expressing cell via intracellular tyrosine kinase activation. At the same time, a reverse signaling cascade is triggered in the Ephrin-expressing cell, via phosphorylation of its intracellular cytoplasmic tail ^18^. Both Ephrins and Eph receptors are membrane anchored proteins and signaling only occurs with cell-cell contact ^19–21^. *EPHRINB2* (*EfnB2*) loss-of-function mutants show early embryonic lethality in mice. However, mutations which only affect *EPHRINB2* reverse signaling result in profound cloacal septation defects and hypospadias, indicating it has a pivotal role in urorectal patterning ^16^.

We have previously described a novel long non-coding RNA (lncRNA) molecule, *Leat1* (long non-coding *EPHRINB2* associated transcript 1), that is essential for female urogenital patterning and development ^22^. Here we describe an essential role for *Leat1* in male urogenital development. *Leat1* directly interacts with EPHRINB2 in the penis and is required for the normal expression of *EfnB2* during urethra closure. Mice lacking *Leat1* display a complete lack of urethral closure leading to severe hypospadias. We further demonstrate that *Leat1* is a target of endocrine disruption in the etiology of hypospadias in both mice and humans.

## Results

### Leat1 expression in the male genital tubercle

**Figure 1.**
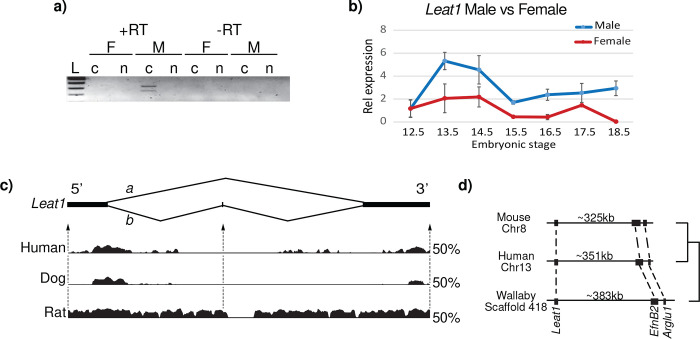
Characterization of *Leat1* **a)** RT-PCR on RNA extracted from male (M) and female (F) genital tubercles at E14.5. *Leat1* was specifically expressed in male and had a predominantly cytoplasmic localization. The two bands indicate that *Leat1* undergoes alternative splicing. c, cytoplasm; n, nucleus. L, 1kb ladder, +RT reverse transcribed template, -RT native RNA template. **b)** Quantitative real-time PCR showing *Leat1* expression in the genital tubercle during development in wild type male (blue solid line) and wild type female (red solid line). In wild type males, *Leat1* reached maximum expression at E13.5, shortly before the onset of urethra internalization. *Leat1* was significantly lower in females throughout this window. **c)** Schematic representation of *Leat1* RNA after sequencing the two products observed in d). *Leat1*a (2 exons) and *Leat1*b (3 exons) 5’ and 3’ end sequences are shown in Supplementary Fig 1c. VISTA Plots show the conservation of *Leat1* sequence across Human, Dog, and Rat relative to Mouse. Exons 1 and 3 tend to be conserved whereas exon 2 was less so. Y-axis represents 50% identity cutoff. **d)** Synteny map showing conservation of relative sequence locations of *Leat1*, *EfnB2*, and Arglu1, *EfnB2*’s closest neighboring gene.

*Leat1* (Long non-coding RNA *EfnB2* Associated Transcript 1, ENSEMBL accession number AK042353), is located approximately 300kb downstream from the *EfnB2* gene ^22^. Amplification of *Leat1* RNA from the E13.5 male genital tubercle identified two isoforms *Leat1a* and *Leat1b* and confirmed that it produces a unidirectional transcript (Fig 1A Supplementary Fig 1A and 1B). Rapid amplification of cDNA Ends (RACE) was used to define the full-length transcripts (Supplementary Fig 1C). We examined the sub-cellular localization of *Leat1* to determine its potential for functional interactions with proteins and/or genomic DNA (Fig 1A. *Leat1* was only detected in the cytoplasmic fraction of male cells from the E14.5 GT. *Leat1* levels were below detection limits in both the cytoplasmic and nuclear fractions of the female GT. Expression of *Leat1* was further examined using more sensitive quantitative RT-PCR (qRT-PCR) in whole GTs during embryonic development to determine its temporal profile. *Leat1* levels peak in males between E13.5 and E14.5 (Fig 1D), directly following the testosterone surge from the developing testis and directly prior to the initiation of urethra internalization. *Leat1* expression decreased at E15.5 before remaining consistent from E16.5 through to E18.5. *Leat1* levels were significantly lower in females throughout this window of development. We also examined the spatial distribution of *Leat1* using whole mount *in situ* hybridization. At E13.5 expression was dispersed throughout the distal aspect of the GT and more concentrated in the urethral plate epithelium (Supplementary Fig 1D–G).

Long non-coding RNAs typically show low sequence conservation between species ^23^, however, VISTA plot analysis revealed extensive conservation of exons 1 and 3 of the *Leat1* transcript across deeply divergent mammalian genomes (Fig 1C). This unexpected conservation strongly suggests that *Leat1* function is sequence dependent. Furthermore, *Leat1* showed positional conservation remaining in synteny with *EfnB2* in human, mouse and even in the distantly related marsupials (which last shared a common ancestor with mice and humans 160 million years ago; Fig 1D) indicating a functional relationship between these genes. In mice, *Leat1* was positioned approximately 325kb (350kb in human and 380kb in wallaby) downstream of the *EfnB2* transcription initiation site.

### Loss of Leat1 causes hypospadias

**Figure 2.**
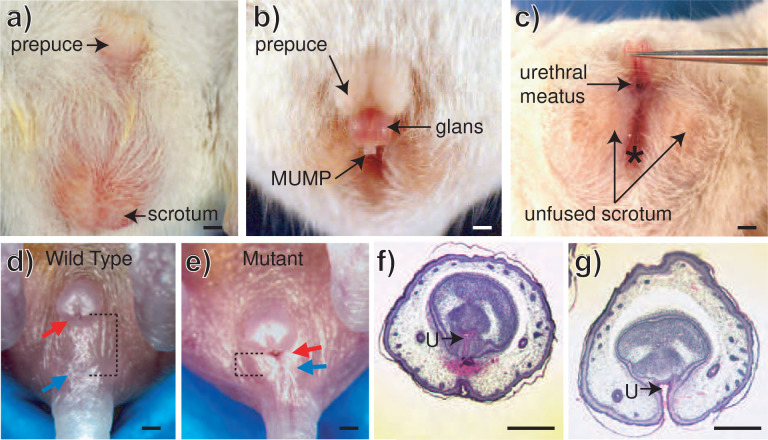
Analysis of the hypospadias phenotype in OVE442 mice. **a)** Gross morphology of the adult wild type male. The penis sits within the abdominal cavity with the external prepuce visible. **b)** Gross morphology of the penis in the mutant mouse line. The penis was externalized and the glans is visible. The prepuce forms a dorsal hood, identical to that seen in human hypospadias. The penis is lifted in **c)** to expose the ventral surface. The urethra remains open along the ventral surface of the penis and the urethral opening (meatus) is located at the base of the phallus. There is a deep groove connecting the urethral opening to the anus (indicated with *) and the scrotum is unfused (bifid) in the midline. **d-g)** The mutant phenotype is clearly observable at birth. **d)** Gross morphology of a wild type male pup on the day of birth. Red arrow indicates the location of the urethral meatus at the tip of the penis and the blue arrow indicates the position of the anus. The dotted line shows the distance between these two structures (anogenital distance). **e)** Gross morphology of the external genitalia in a mutant mouse male pup on the day of birth. The urethral opening is located at the base of the penis (red arrow) and is in close proximity to the anus (blue arrow; dotted lines indicate anogenital distance). **f)** Transverse histological section through the middle of the penis on the day of birth stained with hematoxylin and eosin. In the wild type the urethra (U) is centrally located and closed on the ventral surface. In the mutant **g)** the urethra is open on the ventral surface. Scale bars = 500 μm

An isolated hypospadias phenotype (Fig 2) was identified in mice with a 50kb genomic deletion that spanned the *Leat1* gene ^24,25^ (Supp Fig 1H). No other genes are present within the deletion interval. Adult mutant mice show an unfused scrotum, with reduced anogenital distance (AGD) compared to controls. Mutants had a functional anus, indicating this to be a discrete penis/scrotal malformation and not an anorectal phenotype. In wild type mice, the penis is completely internalized in its flaccid state and the urethral meatus is located at the distal tip of the glans (Fig 2A), whereas in homozygous mutants (Fig 2B,C) the penis was constantly externalized with the meatus located at the base of the glans (Fig 2B,C). The mutant penis also presented a dorsal foreskin hood, identical to the foreskin phenotype seen in human hypospadias cases (Fig 2B). The hypospadias phenotype is also observed at birth in homozygous mutants (Fig 2E) with the urethral meatus located at the base of the penis and the urethra and anus in close proximity (reduced AGD). Histological sectioning shows the urethral plate was formed but not closed in the mutant mouse (Fig 2G).

Since penis development and the process of urethra internalization are androgen regulated, we looked for evidence of androgen deficiency in our mutant mice. Although androgen levels cannot be measured in early developing embryos, we found that all androgen-responsive tissues, including the epididymis, seminal vesicles and os penis, developed normally in the mutant adults (Supplementary Fig 2). This indicates that androgen levels were within the normal range during early development. In addition, the testes developed normally with no sign of hypoplasia. Although spermatogenesis appeared normal in the mutant males, they did not produce offspring because of the physical abnormalities of the penis.

### *Leat1* regulates EPHRINB2 function

**Figure 3.**
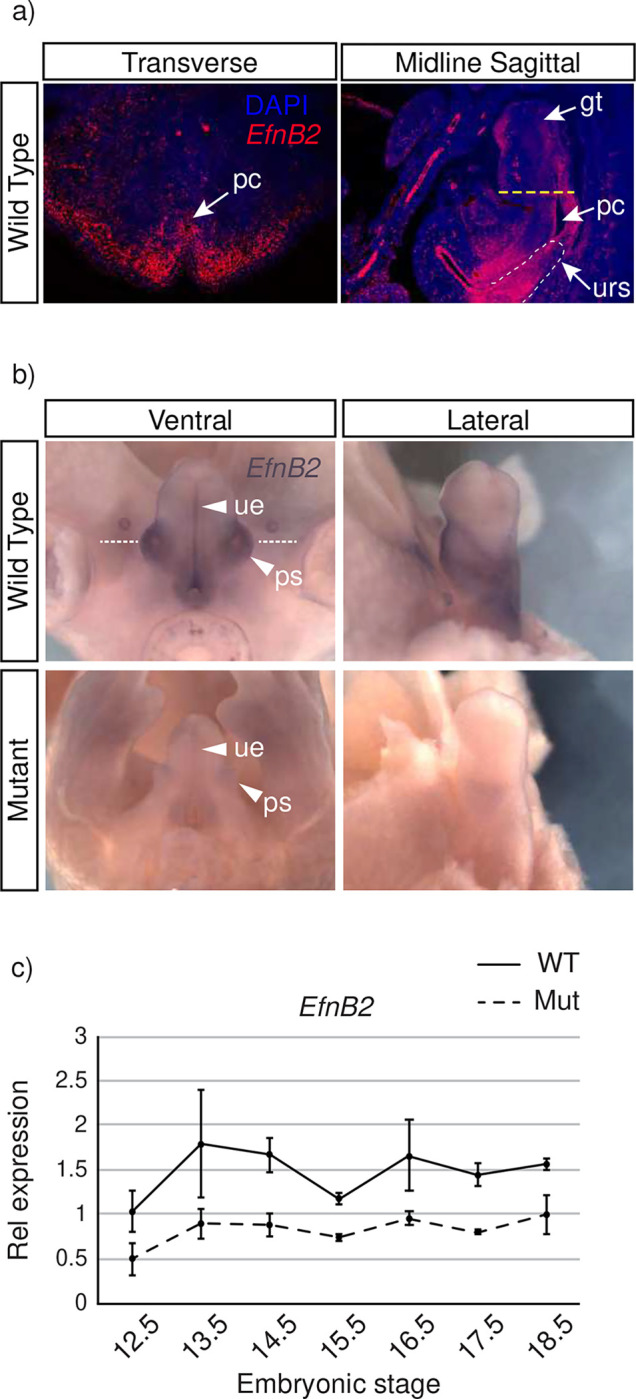
Expression of *EfnB2* in the mouse genital tubercle. **a)** Expression of *EfnB2* in wild type genital tubercle at E14.5 detected by section fluorescence in situ hybridization showing dense *EfnB2* expression throughout the epithelia and mesenchyme. Yellow dotted line shows approximate section depth of the transverse panel. pc, phallic cloaca; gt, genital tubercle; urs, urorectal septum. Scale bar= 200 μM. **b)** Expression of *EfnB2* at E14.5 by whole mount in situ hybridization in wild type and mutant. Ventral and lateral views show strong *EfnB2* expression in the epithelium of the urethra and the preputial swellings (ps). *EfnB2* expression was reduced in the mutant genital tubercle and in the epithelia surrounding the urethra (ue). **c)** Quantitative real-time RT-PCR showing relative expression of *EfnB2* in the wild type (solid line) and mutant (dotted line) genital tubercle throughout embryonic development. *EfnB2* expression was reduced by approximately half in the mutant genital tubercle throughout development, similar to levels seen in the female GT (Supplementary Fig 3b) throughout this window.

The conservation of synteny between *Leat1* and the *EfnB2* gene and its previously reported function in urorectal development ^16,26^, led us to further investigate their relationship. First, we characterized *EfnB2* and its protein product EPHRINB2 in urorectal patterning in wild type embryos (Fig 3A). *EfnB2* mRNA was localized to the surface epithelium and mesenchyme of the male genital tubercle and was more abundant in the epithelium of the urethra, surrounding mesenchyme, and preputial swellings (Fig 3A, Transverse). Sagittal sections show *EfnB2* mRNA localized throughout the epithelium of the urogenital sinus, including the urorectal septum (Fig 3A, Sagittal). These results are consistent with those reported for mRNA localization in the GUDMAP database at E15.5 ^27^. Next, we compared *EfnB2* mRNA expression in male wild type and mutant embryos by whole mount in situ hybridization (Fig 3B). In male wild type embryos, *EfnB2* expression was observed within the preputial swellings, urethral epithelium and in the surrounding mesenchyme (Fig 3B, white arrowheads). *EfnB2* transcripts were substrantially less abundant in the GT of *Leat1* deficient male embryos but similarly distributed (Fig 3B, white arrowheads).

To quantify *EfnB2* downregulation, we performed qRT-PCR to measure mRNA levels in the male GT between E12.5 and E18.5 (Fig 3C). In wild type mice, *EfnB2* expression peaked between E13.5 and E14.5 before decreasing at E15.5, a pattern similar to that observed for *Leat1* expression (See Fig 1B). Homozygous deletion of *Leat1* resulted in a significant decrease in the expression of *EfnB2* (by ~50%) at all stages from E12.5 through E18.5, similar to the levels seen during female GT development (Supplementary Fig 3A). We next investigated the potential regulatory role of *Leat1* for *EfnB2* expression.

### *EfnB2* expression is regulated by *Leat1*

**Figure 4.**
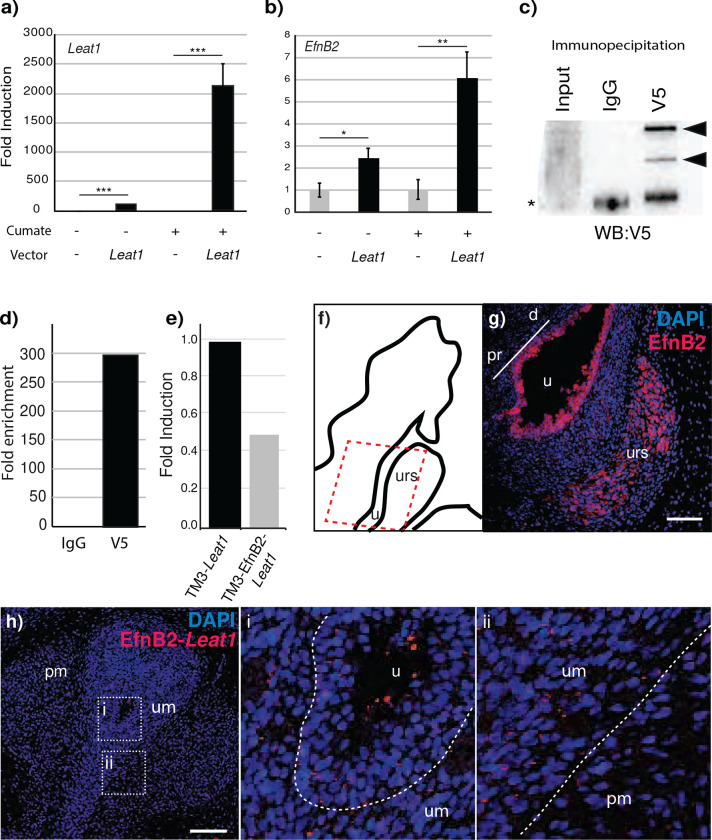
*Leat1* regulates *EfnB2* expression and binds to the EPHRINB2 protein. **a)** TM3 cells expressing cumate-inducible *Leat1* where treated for 48h with cumate to induce *Leat1* expression. RNA expression was quantified using real-time quantitative PCR. We saw a more than 2000-fold induction of *Leat1* (black bars) over non-*Leat1* (gray bars) expressing cells when *Leat1*-positive cells were treated with cumate. *** = p<0.001. **b)** *EfnB2* expression was induced more than 6-fold with cumate induced *Leat1* expression compared to that of non-*Leat1* expressing cells. * = p<0.05; ** = p<0.01. **c)** Western blot showing the efficiency of protein precipitation with the V5 antibody. EPHRINB2 was isolated specifically using the V5 antibody (black arrowheads) when compared to IgG control precipitation. Asterisk = IgG heavy chain. **d)** RNA immunoprecipitation showing *Leat1* binding to the EPHRINB2 protein. TM3 cells expressing inducible *Leat1* and V5-tagged *EfnB2* were treated with cumate for 48h. Proteins were precipitated using the V5 antibody (V5) or control IgG (IgG), RNA extracted and *Leat1* quantified using real-time quantitative RT-PCR. Only protein extracts precipitated using the V5 antibody showed presence of the *Leat1* transcript. Fold enrichment was calculated using the ΔΔCt method ^55^ comparing V5 over IgG signals. **e)** EPHRINB2 can feedback to regulate its own expression. TM3 cells expressing either inducible *Leat1* alone (TM3-*Leat1*) or inducible *Leat1* and constitutive *EfnB2* (TM3-*Leat1*/*EfnB2*) were cultured in the presence of cumate for 48h. Levels of endogenous *EfnB2* were quantified by quantitative real-time RT-PCR. In the presence of exogenous *EfnB2*, levels of endogenous *EfnB2* were reduced to almost 50%, indicating that the exogenous *EfnB2* triggered repression of the endogenous gene. **f)** Schematic indicating plane of section (red outline) of g) through the E17.5 mouse genital tubercle. **g)** Immunohistology for EphrinB2 in saggital section of E17.5 mouse genital tubercle, showing staining in urethral epithelium and the mesenchyme of the URS (Scale = 200 μm; d, distal; pr, proximal; u, urethra; urs, urorectal septum). **h)** Proximity-ligation assay demonstrating direct *in vivo Leat1*-EphrinB2 interaction (red foci) in urethral epithelium (white outline in i) and adjacent mesenchyme (urethral mesenchyme (um) and penile mesenchyme (pm) in ii) of the E17.5 mouse genital tubercle (Scale = 200 μm; u, urethra)).

We used a cell culture system to explore the regulatory relationship between *Leat1* and *EfnB2* expression. The mouse Leydig cell line, TM3, has an epithelial phenotype, is responsive to sex hormones and endogenously expresses *EfnB2* ^28^. We produced a stably transfected TM3 cell line with an inducible full length *Leat1a* transgene under the control of a cumate inducible promoter. Expression of *Leat1a* from the transgene in the absence of cumate was low, but still significantly higher than in cells containing empty vector which showed no endogenous *Leat1* expression (Fig 4A). The expression of *Leat1a* was induced more than 2000-fold by the addition of cumate to the culture medium (Fig 4A). We measured the effect of *Leat1* induction on the expression of endogenous *EfnB2* and observed that *EfnB2* was significantly upregulated (more than 6-fold) when *Leat1* expression was induced (Fig 4B). Interestingly, even in the absence of cumate, the low level induction of *Leat1* expression was still able to significantly increase *EfnB2* mRNA levels. These results demonstrate that *Leat1* regulates *EfnB2* gene expression at the level of transcription and that it can function in this role in trans, outside of its typical genomic context.

### *Leat1* associates with the EPHRINB2 protein both *in vivo* and *in vitro*

Given that *Leat1* showed a predominantly cytoplasmic localization, we examined the possibility of it binding to EPHRINB2 protein. Stable TM3 cell lines containing the *Leat1a* inducible construct were transfected with a V5-tagged-*EfnB2* cDNA. After cumate induction of *Leat1a* mRNA, we used the V5 antibody to precipitate the EPHRINB2 protein along with any interacting RNA. RNA was extracted from the precipitate and qRT-PCR was performed to quantify *Leat1a* mRNA. Immunoprecipitation with control IgG did not isolate any EPHRINB2 protein (Fig 4C) nor show any amplification of *Leat1a* (Fig 4D). When the V5 antibody was used, EPHRINB2 was successfully precipitated (Fig 4C) and we detected *Leat1a* cDNA with a ~300-fold enrichment over IgG alone (Fig 4D).

To demonstrate a physical interaction *in vivo* between *Leat1* and the EfnB2 protein we performed *Leat1*-EfnB2 proximity ligation assays (PLA) on mouse GT tissue at E17.5. Immunofluorescence of EfnB2 on saggital sections showed membrane and cytoplasmic labelling throughout the urethral epithelium and in mesenchyme of the URS (Fig 4F, G). PLA foci are present in the urethral epithelium and concentrated in the adjacent penile mesenchyme (Fig 4 H, i and ii), while no foci were detected with *Leat1* sense probe (Supplementary Fig 4). Due to the low abundance of *Leat1*, this is in line with the amount of staining expected with a Leat1 probe.

Together, these data show that *Leat1* binds the EPHRINB2 protein both in vitro and in vivo, consistent with its predominantly cytoplasmic and membrane localization. The cytoplasmic localization of *Leat1* appears to be facilitated by the presence of a 12bp polyA sequence found at the 3’ end of the transcript - as confirmed by rapid amplification of cDNA ends (RACE) – that is present in the genomic DNA (Supplementary Fig 1C). Coincidentally, 12 adenine residues is the minimum required to bind polyA binding proteins ^29^, to stabilize mRNAs in the cytoplasm.

### *EfnB2* regulates its own expression via a feedback loop involving *Leat1*

Many Ephrins are known to regulate their own expression^30^. To determine whether *Leat1* is involved in the autoregulation of *EfnB2*, we first examined if *EfnB2* can regulate its own expression and second, if this was affected by presence of *Leat1*. We transfected TM3 cells with either the V5 tagged *EfnB2* plasmid or empty vector. Native TM3 cells do not express any endogenous *Leat1*. In the absence of *Leat1*, exogenous *EfnB2* was unable to repress endogenous *EfnB2* expression (Supplementary Fig 3B). This experiment was then repeated in the presence of the cumate inducible *Leat1a* allele described above. There was a significant suppression of mRNA from the endogenous *EfnB2* locus in cells expressing both exogenous *EfnB2* and *Leat1* (Fig 4E).

### *Leat1* is suppressed by estrogen

We investigated if estrogen could affect *EfnB2* and *Leat1* expression in the developing GT. GTs were dissected from wild type and *Leat1*-deficient embryos at E12.5 and cultured for 48h in hanging drop culture in the presence of dihydrotestosterone to drive virilization (as previously described ^31–34^; controls), and with the addition of estrogen (17β-ethinylestradiol; EE2).

**Figure 5.**
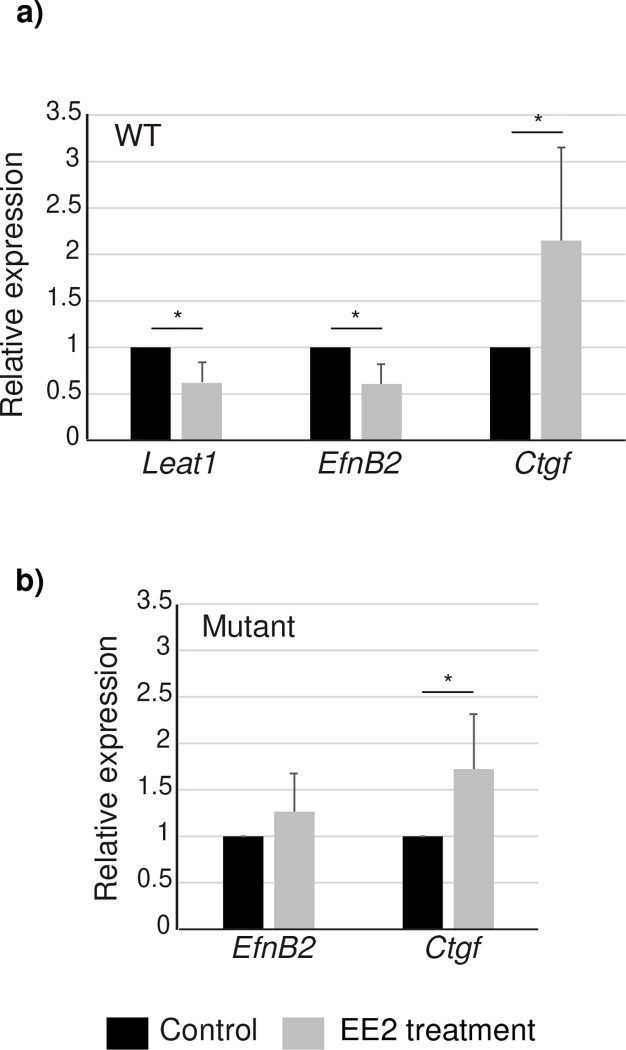
Estrogen regulates *EfnB2* expression via *Leat1*. **a)** Wild type genital tubercles were dissected at E12.5 and cultured over 48h in the absence or presence of 10nM 17β-ethinylestradiol (EE2). After RNA extraction, gene expression was measured by real-time quantitative RT-PCR. Ctgf was used as a positive control of EE2 action. Both *EfnB2* and *Leat1* expression was significantly downregulated by EE2 treatment whereas Ctgf expression increased as expected. **b)** Mutant genital tubercles were dissected at E12.5 and cultured over 48h in the absence or presence of 10nM 17β-ethinylestradiol and expression of *EfnB2* and Ctgf quantified. In the mutant, in the absence of *Leat1*, *EfnB2* expression was unaffected by EE2 treatment. * = p<0.05, grey bars indicate EE2 exposures, black bars = control.

At the end of the culture period, the tissues were snap frozen and gene expression levels examined by qRT-PCR. Connective tissue growth factor (*Ctgf*), a known estrogen responsive gene in the penis ^35,36^, was used as a control to indicate a positive estrogenic response. *Ctgf* was significantly increased compared to controls in both wild type (Fig 5A) and *Leat1* deficient GTs (Fig 5B) cultured with EE2, indicating that the GTs maintained their hormonal responsiveness.

In wild type GTs (Fig 5A), the addition of EE2 significantly decreased both *Leat1* and *EfnB2* expression. *EfnB2* levels were reduced to around half that seen in wild type embryos, similar to that in the *Leat1* deficient mice (which is sufficient to cause hypospadias^26^) and similar to levels seen in the developing female GT (see Supplementary Fig 3A). Significantly, EE2 did not cause a decrease in *EfnB2* expression in the *Leat1* deficient GTs (Fig 5B).

### *LEAT1* is expressed in the human penis

**Figure 6.**
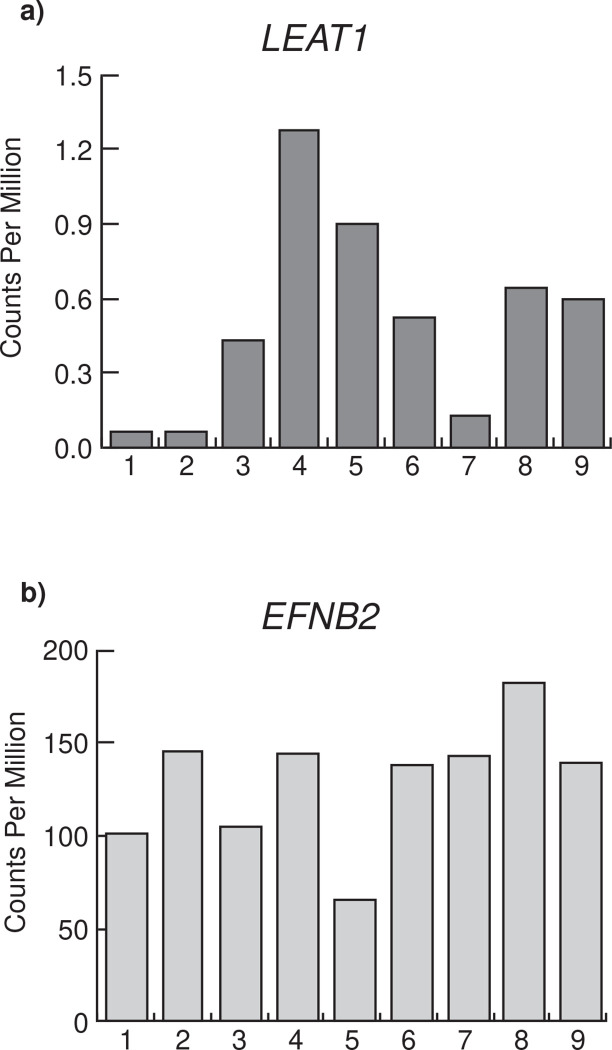
*LEAT1* is expressed in the human penis. Reads were aligned to the hg38 Human genome using the Subread aligner and counted using the featureCount function provided in the Subread package. Read counts were normalized using the CPM function from edgeR. Given that the full length of *LEAT1* is unknown in humans, CPM was used in lieu of other length-bias correcting normalization methods. *LEAT1* counts in Human were made by extracting reads aligned to the homologous region between mouse *Leat1* and Human chromosome 13 from Human RNA-seq UE sample using Samtools. **a)** *LEAT1* expression levels in urethral plate epithelium of patients presenting with mild hypospadias. **b)** *EFNB2* expression levels in urethral plate epithelium of patients presenting with mild hypospadias.

As described above, *LEAT1* is unusually conserved for a lncRNA. Orthologous sequences were readily detected by shared homology across all mammals and always in synteny with *EFNB2*, including in the human genome (Fig 1C and 1D). We next determined if *LEAT1* was a functional gene in humans and capable of producing mRNA transcripts in the human penis alongside *EFNB2* ^37,38^. We examined *LEAT1* and *EFNB2* mRNA levels in the transcriptomes of urethral plate epithelium (UPE) isolated from patients undergoing repair surgery for mild hypospadias (Fig 6). Although the timing of tissue collection was after the window of urethra internalization in humans, *LEAT1* expression was detected in the UPE of the human patient samples (Fig 6A). Interestingly, *LEAT1* expression levels were variable between samples and very low in 3 out of the 9 hypospadias patients. *EFNB2* was also expressed in the human UPE, as it is in mice, and showed variable levels across patient samples, however, there was no correlation between *LEAT1* and *EFNB2* expression levels (Fig 6B). These data demonstrate that the human *LEAT1* locus produces an mRNA transcript that is expressed in the urethral plate of the penis alongside *EFNB2*, as in mice.

## Discussion

*Leat1* is a novel, hormonally regulated lncRNA which binds to and facilitates EfnB2 function. Deletion of *Leat1* leads to a suppression in *EfnB2* expression and a complete lack of urethral closure resulting in hypospadias. In addition, exogenous estrogen supresses *EfnB2* expression in a *Leat1* dependent manner. Finally, we show that *LEAT1* was conserved in humans and produces a mRNA that was co-expressed alongside *EFNB2* in the human penis. Together, our *in vitro* and *in vivo* data identify a new, hormonally regulated driver of urethra internalization that sits at the interface between the genome and the environment in the development of hypospadias.

### *Leat1* binds to and regulates *EfnB2* and is required for urethral closure

We have shown that *Leat1* mRNA physically binds the EPHRINB2 protein both *in vitro* and *in vivo* in the developing penis. This is consistent with the predominantly cytoplasmic localization of *Leat1* and its functional poly-A tail. EPHRINB2 sits in the plasma membrane ^17^ and has a large intracellular tail which is the only region to which cytoplasmic *Leat1* could bind. To explore how *Leat1*-EPHRINB2 binding might affect *EfnB2* gene regulation we examined the impact of this interaction on *EfnB2* autoregulation. In the presence of *Leat1*, exogenous *EfnB2* overexpression was able to suppress transcription of the endogenous *EfnB2* gene. However, in the absence of *Leat1*, *EfnB2* was unable to autoregulate. Therefore, *Leat1* likely mediates *Efnb2* autoregulation in the developing penis where both genes are co-expressed.

Loss of *Leat1* in mice caused a complete lack of urethral closure, observable from birth and persisting through to adulthood. The location of the urethral meatus at the base of the penis in homozygous mutant mice, in conjunction with the unfused scrotal bulges, reduced AGD and dorsal foreskin hood, corresponds to the clinical classification of a severe hypospadias phenotype in humans (Fig 2B) ^39^. The *Leat1* deficient mouse phenotype was remarkably similar to that of heterozygous mice with a mutant form of *EfnB2* (the EfnBlacZ allele;^16^). In these mice, the cytoplasmic domain of the EPHRINB2 protein was replaced by a *lacZ* cassette, resulting in a fusion protein lacking reverse signalling ^16^. EPHRINB2 reverse signalling is known to regulate epithelial to mesenchymal transitions, a likely critical pathway in mediating the internalization of the urethra ^40–42^. *EfnB2* expression was significantly lower in *Leat1* homozygous mutants. Given the defined role of EPHRINB2 in urorectal patterning, the phenotype observed in *Leat1* mutants is consistent with hypomorphic expression of *EfnB2* being the causal factor.

### *Leat1* is a potential target of endocrine disruptors in the etiology of hypospadias

*Leat1* expression was sexually dimorphic and peaks in the male GT at E13.5, directly after the onset of androgen production from the fetal Leydig cells ^43^. We next investigated the impact of estrogen on *Leat1* and *EfnB2* expression in the developing GT during the window of urethral closure. Male GTs cultured in the presence of DHT showed normal urethral colure and normal upregulation of *Leat1*. In contrast, GTs exposed to estrogen and DHT showed a significant reduction in the expression levels of both *Leat1* and *EfnB2* to around half of that seen in the normal GT. This was equivalent to *Leat1* and *EfnB2* expression levels in the female GT (Fig 1B, Supplementary Fig 3A) and an equivalent level of suppression of *EfnB2* to that seen in the *Leat1* null mutant *in vivo* (Fig 3C). Furthermore, our GT culture system demonstrates that *Leat1* (and *EfnB2*) are directly supressed by estrogen in the developing penis itself. This suggests EDCs can directly target genes regulating urethral closure in the penis to cause hypospadias, rather than indirectly impacting androgen output from the developing testis ^44^. In addition, we showed that the suppression of *EfnB2* by estrogen was *Leat1* dependent.

### *EfnB2* was not supressed by estrogen in the developing GTs from *Leat1* null mutants.

Estrogens and estrogen-mimicking endocrine disruptors such as BPA and genistein are well known to increase the risk of hypospadias in mice and humans ^11,45,46^. However, the molecular targets of these chemicals are largely unknown. The impact of estrogen on *Leat1* expression implicates it as a direct target of EDCs in the development of hypospadias in human patients. Estrogen exposures do not typically induce severe hypospadias phenotypes, but more mild manifestations of the disease. Exposure to estrogen during later stages of urethra internalization could cause a mild reduction in *Leat1* and subsequently *EfnB2* expression, resulting in mild distal forms of hypospadias. Alternatively, a more potent and early estrogenic exposure during GT development could result in the more severe proximal forms such as that observed in our model with *Leat1* deficiency.

### *Leat1* conservation suggests a critical function in mammalian genital tubercle development

Long non-coding RNAs are typically poorly conserved at the nucleotide level and only identifiable between species based on their genome location or secondary structure ^47–49^. However, *Leat1* was uncharacteristically conserved across mammals with blocks of high nucleotide conservation between mouse, human and even distantly related marsupial mammals (Supplementary Fig 5). In each case, *Leat1* was located in an identical genomic location syntenic with *EfnB2* (Fig 1D). Marsupials last shared a common ancestor with mice and humans 160 million years ago ^50^ indicating an extremely conserved function for *Leat1* in mammals that is both location and sequence dependent. Given that *Leat1* can bind the EPHRINB2 protein, we suggest that the high degree of nucleotide conservation may be important for mediating this interaction. Despite being syntenic with *EfnB2*, *Leat1* overexpression in trans can still drive *EfnB2* upregulation, indicating that *Leat1* can regulate *EfnB2*, even outside of its genomic context ^51^.

We demonstrated that *LEAT1* is a functional gene in humans, producing an mRNA that was expressed in the developing penis alongside *EFNB2*. Interestingly, there was variable *LEAT1* expression in the UPE of humans with mild hypospadias and an almost complete absence in one third of the cases examined (Fig 6). This, together with the sequence conservation of *LEAT1*, suggests a conserved interaction with EPHRINB2 to mediate urethra internalization and implicates it as a potential candidate gene in human hypospadias.

### A model for *Leat1* function in urethra internalization

**Figure 7.**
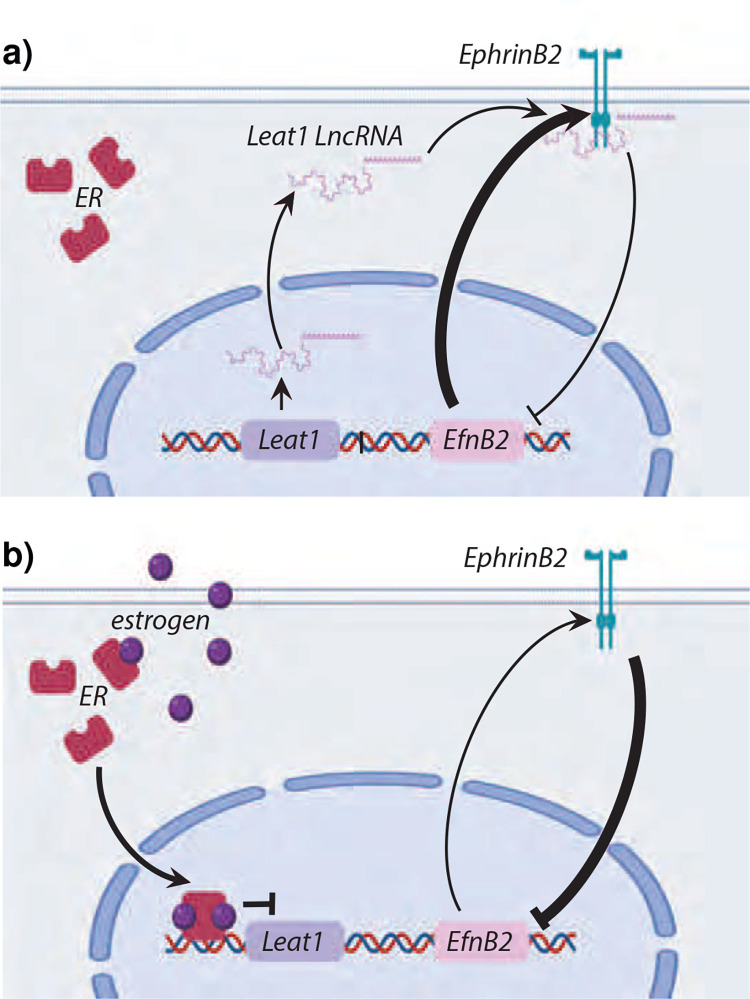
Model of *EfnB2* regulation during mouse urethra formation. **a)** *EfnB2* expression is maintained by a feedback loop in which membrane-bound EPHRINB2 signals back to the *EfnB2* gene to regulate its expression. At the onset of urethra internalization, *Leat1* transcripts are upregulated and bind to the EPHRINB2 protein inhibiting the negative feedback loop resulting in elevated *EfnB2* levels. **b)** In the absence of *Leat1* (as seen in our mutant mouse line) or following its suppression by estrogen, the EPHRINB2-*EfnB2* negative feedback loop is no longer inhibited. As a result, *EfnB2* levels remain low and urethra internalization is not initiated resulting in hypospadias. Arrows indicate known interactions and effects as defined from our data. Thickness of the arrows indicates the strength of the upregulation (arrow) or inhibition (blunt ended arrow). The precise nature of interactions occurring at the nodes of each arrow is speculative.

We have shown that *Leat1* binds to the EPHRINB2 protein *in vitro*. We further demonstrate that *Leat1* binding affects the EPHRINB2 autoregulatory feedback loop in the developing GT. *Leat1* binding reinforces the EPHRINB2 feedback loop, leading to an upregulation of *EfnB2* in the genital tubercle (Fig7A). During normal penis development, the upregulation of *Leat1* at E12.5-E13.5 in males, before the onset of urethra internalization, establishes EPHRINB2 autoregulation leading to expression of *EfnB2* mRNAs to the level necessary for urethra internalization (Fig 1B, Fig 3C, Fig 7A). A loss of *Leat1* expression reduces EPHRINB2 feedback, downregulating *EfnB2* mRNA expression. This, in turn, prevents urethral closure, resulting in hypospadias. Exposure to exogenous estrogen during this developmental window can reduce *Leat1* expression, leading to inhibition of *EfnB2* mRNA below required levels resulting in hypospadias (Fig 7B). Together these data demonstrate an essential regulatory mechanism required for normal urethral closure, and one which can be impacted by exogenous estrogen to cause hypospadias in both mice and humans.

### Conclusions

In conclusion, we have identified a novel lncRNA *Leat1* that regulates urethral closure in mice. *Leat1* regulates the function of its neighboring gene *EfnB2* by affecting its autoregulatory feedback through protein-RNA interactions. This relationship between *Leat1* and *EfnB2* was demonstrated both *in vivo* and *in vitro*. *Leat1* shows extraordinary sequence conservation for a lncRNA, indicating conservation of domains we propose mediate its binding to EPHRINB2. We further show that *Leat1* expression was supressed by estrogen and that this mediates the suppression of *EfnB2*. Importantly, we showed that *LEAT1* and *EFNB2* are also co-localised in the developing human penis. Together our data provide significant new insights into both the developmental mechanisms and molecular regulation of urethra internalization and implicate *Leat1* as a potential target of endocrine disruption in the etiology of hypospadias in mice as well as humans.

## Methods

### Mice

The OVE442 mouse line was obtained from P.A. Overbeek laboratory (Baylor College of Medicine, Houston, USA) and was maintained on a FVB/NJ genetic background ^22^. Wild type FVB/NJ mice were obtained from Monash University Central Animal Services (Melbourne, Australia). Mouse lines were bred and maintained in the Bioscience 4 animal facility. Mouse embryos were collected from timed matings with noon of the day on which the mating plug was observed designated E0.5 (0.5 days post coitum). Protocols and use of animals conformed to the National Health and Medical Research Council/Commonwealth Scientific and Industrial Research Organization/Australian Agricultural Council Code of Practice for the Care and Use of Animals for Experimental Purpose (2004) and were approved by the University of Melbourne Committee on Ethics in Animal Experimentation under the reference 1312882.4.

### Cell lines

TM3 cells were obtained from ATCC and maintained in culture in DMEM + Glutamax (Life Technologies, Sydney, Australia) supplemented with 10% fetal bovine serum (Life technologies), antibiotic and antimycotic solution (Life technologies, Sydney, Australia) at 37°C in 5% CO_2_. Stable cell lines were cultured as above but in media supplemented with the appropriate selective antibiotics.

### Stable cell lines

TM3-PiggyBac *Leat1*a stable cell lines were generated to allow monitoring of gene expression after induction of *Leat1*a expression by treatment with cumate. To produce TM3-PiggyBac *Leat1*a stable cell lines, TM3 cells were seeded in 6-well plates and transfected with 3 μg of PiggyBac-*Leat1*a plasmid together with 1 μg of Super PiggyBac transposase plasmid using Fugene 6 transfection reagent (Promega, Sydney, Australia). 24h after transfection culture media was supplemented with 4 μg/ml puromycin to select for positive clones. To produce TM3-PiggyBac Control stable cell lines, TM3 cells were seeded in 6-well plates and transfected with 3 μg of empty PiggyBac Cumate Switch inducible vector (SBI, Palo Alto, CA) plasmid together with 1 μg of Super PiggyBac transposase plasmid using Fugene 6 transfection reagent (Promega, Sydney, Australia). 24h after transfection culture media was supplemented with 4 μg/ml puromycin (Life technologies, Sydney, Australia) to select for positive clones.

To produce TM3-PiggyBac *Leat1*a/pc*EfnB2* stable cell lines, a TM3-PiggyBac *Leat1*a cell line was transfected with 1 μg of pcDNA-V5-*EfnB2* using Fugene 6 transfection reagent (Promega, Sydney, Australia). 24h after transfection culture media was supplemented with 0.4 μg/ml Geneticin (Life technologies, Sydney, Australia) to select for positive clones. When indicated cumate treatment was performed for 48h at a concentration of 30 μg/ml (1X) in culture medium.

### Genome mutation analysis

We mapped the site of the transgene insertion in the mutant mice to chromosome 8 using standard methods ^25^. The transgene insertion event caused a 50kb deletion in a coding-gene deficient region and was located approximately 300kb downstream from the *EfnB2* gene (Supplementary Fig 1H. We used genomic PCR to verify the transgene insertion boundaries and show that the associated deleted region was specific to our mutant line compared to the background FVB/NJ mice (Fig 1I). The transgene boundaries were identified using inverse PCR according to published methods ^25^. Genomic DNA was extracted and purified using phenol-chlorophorm-isoamyl alcohol DNA extraction. The genomic DNA sequences were amplified with MyTaq^™^ DNA Polymerase kit (Bioline, Sydney, Australia). Primer pair B1 was used for mapping the 5’ end of the transgene insertion boundary and primer pair B2 used for mapping the 3’ of the transgene insertion (Supplementary table 1). PCR products where purified using QIAquick PCR Purification kit (Qiagen, Sydney, Australia) and sequenced by the CTP Sanger Sequencing Service in the Department of Pathology, The University of Melbourne.

### Genital tubercle culture

Genital tubercles were dissected from WT and *Leat1* mutant male embryos at E12.5. GTs were cultured in carbogen using the hanging drop method in BGJb medium (Life Technologies, Sydney, Australia) supplemented with 3 U/ml bovine insulin (Sigma, Sydney, Australia), 0.1 mg/ml L-ascorbic acid (Sigma, Sydney, Australia), 100 U/ml penicillin and 100 U/ml streptomycin (Life Technologies, Sydney, Australia). 10 nM 5α-Androstan-17β-ol-one (DHT; Sigma, Sydney, Australia) and 10 nM 17βethinylestradiol (EE2; Sigma, Sydney, Australia) were dissolved in 100% EtOH. Explants were cultured wither with 10 nM DHT or with 10 nM DHT and 10 nM EE2. Previous studies have shown that in similar culture systems 10 nM DHT is required for induction of GT elongation, prepuce formation and cellular proliferation. Similarly, 10 nM EE2 induces changes in urethral closure without causing excessive growth suppression and altered differentiation evident at dosages higher than 50 nM EE2 ^34^. After 48 hours, gene expression was analyzed by quantitative RT-PCR (see below).

### Plasmid construction

*Leat1* cDNA was amplified by PCR from E14.5 mouse genital tubercle cDNA, using primers Clm353F and Clm353R (Supplementary table 1), encompassing 2159 bp of *Leat1* RNA. The PiggyBac-*Leat1*a inducible expression vector was generated by cloning *Leat1*a cDNA into the NheI and NotI restriction site of the multiple cloning sequence of the inducible PiggyBac vector. pcDNA-V5-*EfnB2* was generated using the pcDNA3.1 directional TOPO expression kit (Invitrogen, Sydney, Australia) following manufacturer instructions. The *EfnB2* ORF was amplified by PCR from E14.5 mouse genital tubercle cDNA using primers Clm*EfnB2*F and Clm*EfnB2*R and subcloned into pcDNA3.1D/V5-His-TOPO^®^ vector. pGem-*EfnB2* was generated using the pGEM^®^-T Easy Vector Systems kit (Promega, Sydney, Australia) following manufacturer instructions. A 736bp fragment of *EfnB2* was amplified from E14.5 mouse genital tubercle cDNA using primers Clim*EfnB2*F and Clim*EfnB2*R and subcloned into pGEM^®^-T Easy vector. All plasmids were sequenced by the CTP Sanger Sequencing Service in the Department of Pathology, The University of Melbourne.

### Genotyping and DNA sequencing

To determine genotypes of OVE442 mice, tail tip (fetus and neonates) or ear notches (adults) were used for genomic DNA extraction using DNeasy Blood and Tissue kit (Qiagen, Sydney, Australia). The genomic DNA sequences were amplified with MyTaq^™^ DNA Polymerase kit (Bioline, Sydney, Australia) using multiplex primers, OVEMutFwd, OVEMutRev, OVEWTFwd and OVEWTRev detecting wild type and mutant DNA (Supplementary table 1). Amplicons were sequenced by the CTP Sanger Sequencing Service in the Department of Pathology, The University of Melbourne.

### In vitro transcription

pGem-m*EfnB2* plasmid was used as template for PCR amplification using T7 and SP6 primers (Supplementary table 1). The amplicon was purified using the QIAquick PCR Purification kit (Qiagen) and used as a template for transcription and labeling using the DIG RNA labeling Kit (SP6/T7) (Roche, Sydney, Australia). SP6 transcription produced antisense probe whereas T7 transcription produced sense probe (control).

### Whole mount in situ hybridization

Mouse embryos collected at E14.5 were fixed in 4% paraformaldehyde (PFA) in PBS for 24 hours at 4°C. Whole mount in situ hybridization (WISH) was carried out as described previously ^52^. Probe signal detection was performed using an anti-digoxigenin antibody from sheep, conjugated with alkaline phosphatase (Roche). Imaging was performed on an Olympus SZX16 microscope equipped with a Nikon DS-Fi2 Digital Sight Camera and using NIS Element software (Nikon).

### Section in situ hybridization

Penises from mice on the day of birth were fixed in 4% PFA overnight, washed twice in PBS and processed for embedding in paraffin wax. 5 μM sections were taken and used for in situ hybridization, performed according to the manufacturer’s protocol (Quantigene ViewRNA ISH Tissue kit, Affymetrix). The *EfnB2* probe was synthesized by Invitrogen based on the mRNA NCBI Reference Sequence Gi: 158508443, Ref: NM_010111.5.

### Section Histology

Genital tubercles were fixed in 4% PFA overnight and washed in PBS and stored in 70% ethanol until processing in paraffin. After processing and embedding, paraffin embedded tissues were serially sectioned with a section thickness of 7 μm. Tissue for section histology was dewaxed in histolene and counterstained with Lillie-Mayer hematoxylin and eosin according to standard methods ^53^.

### Tissue proximity ligation assay (PLA)

Tissue PLA was performed following the protocol of ^54^with minor modifications. Sections of 5um thickness were dewaxed by 2 five-minute immersions in histolene and 100% ethanol, followed by five-minute immersions in decreasing concentrations of ethanol (90%, 70% and 50%) before immersion in distilled H_2_O for five minutes. Tissue was heat-treated by immersion in 10mM sodium citrate buffer with 0.05% tween-20 (pH 6.0), in a waterbath at 95oC for 30 minutes. After washing in distilled H2O for five minutes, tissue was incubated in 0.1M HCl at 37oC for 10 minutes before immersion in distilled H2O for 5 minutes. Tissue was subsequently equilibrated with ISH buffer (2x SSC, 0.05% tRNA, 0.2% BSA in PBS) for 5 minutes at room temperature. 100nM of Biotinylated *Leat1* probes (Integrated DNA technologies: antisense: GGGAATAAAAGCGGGGACTAGACCTTCTGCCTAAAAATAGTCAAT; sense: TGCTATCGTGAATCGGATATTAGTCGTTAACGAGACGCTCCTTGA), were applied to tissue and incubated overnight at 37oC. After incubation with Leat1 probe, tissue was washed twice in 2x SSC and blocked for 30 minutes with 10% normal donkey serum in PBS. After 1 five-minute PBS wash, tissue was incubated with anti-EfnB2 (#ab131536, Abcam) and anti-biotin (#ab201341, Abcam) primary antibodies diluted in PBS with 10% normal donkey serum, for 1 hour at room temperature, followed by an additional 2 five-minute PBS washes. Next, plus and minus oligonucleotide-labelled PLA antibodies were applied (#DUO92001 & #DUO92005, Thermo Fisher Scientific), ligation and polymerization performed (#DUO92007, Thermo Fisher Scientific) following the manufacturers instructions. Sections were counterstained with DAPI (100nM) for 10 minutes at room temperature. Tissue was imaged on a Nikon A1R confocal microscope. Some non-specific reactivity is present in apical urethral cells and blood vessels in sections that received sense probe, and this is not considered to be part of the PLA signal.

### Nuclear and Cytoplasmic RNA extraction

Nuclear and cytoplasmic RNA were extracted from E14.5 genital tubercles using the Cytoplasmic and Nuclear RNA Purification kit (Norgen Biotek Corp. ON, Canada). Tissues were frozen in liquid nitrogen and ground using mortar and pestle. RNA was extracted following the manufacturer’s instructions. RNA was eluted in 40 μl elution buffer and yield was approximately 200 ng/μl for cytoplasmic RNA fraction and 40 ng/μl for nuclear RNA fraction. RNA concentrations were determined using NanoDrop ND-1000 Spectrophotometer. cDNA was prepared from 200 ng of RNA using Random Primers and the SuperScript III First Strand Synthesis System (Invitrogen, Sydney, Australia). cDNA was amplified with MyTaq^™^ DNA Polymerase kit (Bioline) using primers, PCR-F and PCR-R detecting both *Leat1*a and *Leat1*b isoforms.

### RACE analysis of *Leat1* transcript

Rapid amplification of cDNA ends (RACE) was performed using the SMART RACE 5’/3’ kit protocol from Invitrogen. Total RNA was extracted from E13.5 genital tubercles and used for RACE amplification following the manufacturer’s instructions. PCR products were purified using QIAquick PCR Purification kit (Qiagen, Sydney, Australia) and sequenced by the CTP Sanger Sequencing Service in the Department of Pathology, The University of Melbourne.

### Quantitative real-time RT-PCR

Genital tubercles were dissected from male and female embryos from both wild type and *Leat1* mutants at embryonic stages E12.5 through to E18.5 (n=4 for each stage). RNA extractions were performed using the GenElute Mammalian Total RNA Kit (Sigma, Sydney, Australia). RNA concentrations were determined using NanoDrop ND-1000 Spectrophotometer. cDNA was prepared from 800 ng of total RNA using Random Primers and the SuperScript III First Strand Synthesis System (Invitrogen, Sydney, Australia). qPCR was performed on a Stratagene MX300P using QuantiTect SYBR Green (Qiagen, Sydney, Australia). *Leat1* primers were designed to amplify exon 1 of the gene, detecting both *Leat1*a and *Leat1*b. Actin and Hprt were used as housekeeping genes for normalization (Supplementary table 1). Relative quantification of gene expression was calculated according to the Pfaffl method ^55^.

### Analyses of *LEAT1* expression in human urethral plate epithelium samples

A small piece of urethral plate epithelial tissue was collected from patients with mild hypospadias phenotypes during surgical repair. Repair surgeries were conducted between 6 months and 16 months post-partum. Samples were collected under ethics application HREC 35189, The Royal Children’s Hospital Melbourne. RNA was extracted using the Qiagen RNeasy kit and concentrated using the Qiagen MinElute cleanup kit (Qiagen, Sydney, Australia). Libraries were constructed and sequenced at the Flinders Genomics Facility, Flinders Medical Centre, South Australia using the TruSeq Stranded mRNA Library Prep and sequenced on an Illumina HiSeq with >160 million reads per sample for the detection of low-expressed transcripts such as lncRNAs including *LEAT1*.

### RNAseq data was assessed for quality using FastQC

(http://www.bioinformatics.babraham.ac.uk/projects/fastqc). Reads were aligned to the hg38 Human genome using the Subread aligner. Ten bases were trimmed from both read ends prior to alignment and only uniquely mapping reads were returned from the alignments. Read counts were performed using featureCount available from the Subread package for R. Only primary alignments with a mapping quality greater than 10 were considered. *LEAT1* counts in human were performed by extracting reads aligning to the *LEAT1* locus on Chromosome 13 using Samtools. The resulting counts were concatenated to the featureCount data prior to normalization. For within-sample expression quantification, counts per million (CPM) were calculated using the edgeR package. The full *LEAT1* transcript length is unknown in humans so other length correction normalization methods were considered inappropriate.

### RNA Immunoprecipitation

RNA protein immunoprecipitation was performed using a Magna RIP kit (Millipore, Sydney, Australia). Briefly, stable TM3 cell lines containing the *Leat1*a inducible construct were transfected with a V5-tagged-*EfnB2* cDNA. Cells were treated with cumate (30μg/ml) to induce *Leat1* expression. After 48h, 1×10^7^ cells were washed with ice-cold PBS and lysed on ice with RIP lysis buffer. Protein G magnetic beads (Dynabeads, ThermoFisher Scientific, Sydney, Australia) with antibodies against the V5 tag (ThermoFisher Scientific, Sydney, Australia) or control IgG, were incubated with the cell lysate overnight at 4°C. Precipitated V5-EPHRINB2 protein or control precipitate were incubated with 10% SDS and proteinase K and the supernatant was used for RNA extraction. After RNA purification and precipitation, RNA was resuspended in 30 μl of RNase-free H_2_O. 10 μl of RNA was used for qRT-PCR.

### Immunoprecipitation

Stable TM3 cell lines containing the *Leat1*a inducible construct were transfected with a V5-tagged-*EfnB2* cDNA. Cell were lysed with RIPA buffer (50 mM Tris HCl, pH 8.0, 170 mM NaCl, 0.5% Nonidet P-40, 0.5% Sodium deoxycholate, Complete protease inhibitors). Cell lysates were centrifuged at 14,000 × g for 10 minutes, and supernatants were used for immunoprecipitation. After preclearing for 1 hour with dynabeads protein G (ThermoFisher Scientific, Sydney, Australia), supernatants were incubated at 4°C overnight with antibodies against V5 tag or control IgG and 50 μg of protein G Dynabeads. Immunoprecipitates were washed five times with lysis buffer, and proteins were eluted with SDS loading buffer, resolved on SDS Page gel and analyzed by immunoblotting using the antibodies as indicated.

## Supplementary Material

Supplement 1
