# Supplementary material for: A long non-coding RNA Leat1 mediates the hormone responsiveness of EfnB2 during male urogenital development
Source: Res Sq. 2023 Jul 3:rs.3.rs-3098271. Preprint. [Version 1] doi: 10.21203/rs.3.rs-3098271/v1 (PMC10350214; doi:10.21203/rs.3.rs-3098271/v1)
Supplement: Supplement 1 [file NIHPPrs3098271v1-supplement-1.pdf]

Supplemental Data

Supplemental Fig 1. Characterization of *Leat1* transcript. **a)** Schematic representation of *Leat1* transcripts. Black arrowheads represent *Leat1* primers used in this study. F1, F2, R1, R2 were used to determine the orientation of the RNA in b). PCR-R and PCR-F represent primers used to detect *Leat1* in nuclear and cytoplasmic fractions. qPCR-F and qPCR-R show positions of primers used in the qPCR experiments. **b)** Agarose gel electrophoresis of RT-PCR products from amplification using primer sets depicted in a) showing that *Leat1* is transcribed exclusively from the plus strand of genomic DNA. 2log shows the 2log DNA ladder, RT Neg shows PCR on native RNA, Neg shows template minus control. **c)** Sequence of the *Leat1* clone, obtained after RACE PCR (*Leat1* Clone) showing alignment to the genome (Genome) and cDNA clone from NCBI database (ucsc_ak042353.1) (https://www.ncbi.nlm.nih.gov/nuccore/AK042353). Exon 2 sequence is not present in clone ucsc_ak042353.1. We defined an additional 6bp of open reading frame at the 5' end of the transcript compared with ucsc_ak042353.1 and a polyA tail encoded in the genome and present in the transcript at the 3' end. Panels D-G show whole mount in situ hybridization of *Leat1* in the male GT at E13.5. **d)** Lateral view of the GT showing *Leat1* staining (dark blue) in the distal aspect of the developing penis (arrow). **e)** Ventral view of the same GT showing concentrated staining along the urethral plate epithelium (arrow). **f)** Expanded view of the UPE to show detail of staining and black and white contrast rendering of the staining to show the distribution pattern. **g)** *Leat1* in situ staining is consistent with the shape of the urethral plate at E15.3 (arrows) shown by sagittal section histology. **h)** Schematic representation of the transgene insertion in the mutant mice (Mutant) compared to wild type. The OVE442 mutation resulted in a loss of approximately 50kb of genomic DNA on chromosome 8 with at least 2 copies of transgene insertion. The genomic rearrangement was located approximately 300kb downstream of the *EfnB2* termination sequence. Within the deleted region, only *Leat1* was entirely removed. **i)** Confirmation of location of transgene insertion and genomic deletion by PCR. B1 and B2 primers were designed at the boundary of the transgene insertion. The FVB/NJ strain, the parent strain of OVE442 mice, was used as negative control. The deletion interval was detected only in OVE442 but not FVB/NJ. Neg, no DNA control; L, 1kb ladder.

Supplemental Fig 2. Gross morphology of the internal reproductive tract of wild type and *Leat1* mutant adult males. The highly androgen sensitive seminal vesicles (black arrowhead), epididymides and overall testis size (*) were similar between wild type and mutant mice indicating normal virilization.

Supplemental Fig 3. *Leat1* regulation of *EfnB2*. **a)** Quantitative real-time RT-PCR showing relative expression of *EfnB2* in the male wild type (blue solid line), male mutant (blue dotted line) and female wild type (red solid line) genital tubercle throughout embryonic development. *EfnB2* expression was reduced by approximately half in the male mutant genital tubercle throughout development, similar to levels seen in the female GT throughout this window. **b)** *EfnB2* autoregulation was reduced in the absence of *Leat1*. TM3 cells, which do not express endogenous *Leat1*, were transfected with an empty pcDNA control vector (-) or with pcDNA-V5-*EfnB2*. After 48h, RNA was extracted and levels of endogenous *EfnB2* quantified by quantitative real-time RT-PCR.

Supplemental Fig 4. *Leat1*-EfnB2 proximity ligation assay (PLA) on mouse GT tissue at E17.5 with a *Leat1* sense probe. **a)** No foci were detected in either the uethral epithelium or the surrounding penile mesenchyme (pm). **b)** Foci within the urethra are non-specific staining (d, distal; pr, proximal; u, urethra).

Supplemental Fig 5. *Leat1* nucleotide sequence is conserved across species. Multiple sequence alignment for Mouse, Human and Wallaby *Leat1* nucleotide sequences were performed using T-COFFEE [56].

Supplemental Table 1. Primers used for the study.

a)

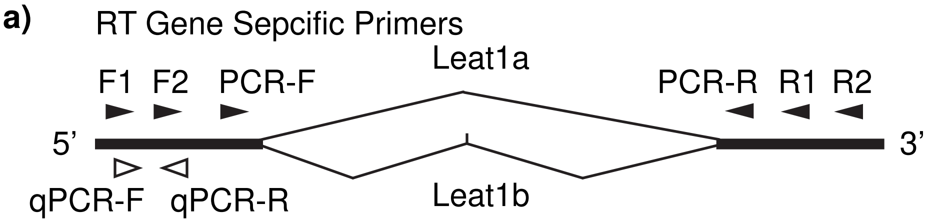

b)

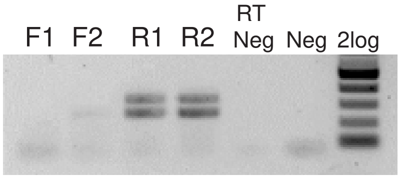

c)

5' End [Exon 1; 5' start of transcript defined by RACE]

```
Leat1 Clone       5' - AGACTCAAGCTTTGCCTCCCGCTGGAAAAGGACCTGGGTCACA - 3'
ucsc_ak042353.1   5' - ------AAGCTTTGCCTCCCGCTGGAAAAGGACCTGGGTCACA - 3'
Genome            5' - AGACTCAAGCTTTGCCTCCCGCTGGAAAAGGACCTGGGTCACA - 3'
                             *************************************
```

Exon 2

```
Leat1 Clone       5' - --CTGCTTGACTGGCTGAATTGCTTCTCCTGCAGGCTTGACTG
ucsc_ak042353.1   5' - -------------------------------------------
Genome            5' - ACCTGCTTGACTGGCTGAATTGCTTCTCCTGCAGGCTTGACTG
                         *****************************************

Leat1 Clone            GCATGTTTCCAGAGAAGACCTGCTGGAGCTGGGGG-------- - 3'
ucsc_ak042353.1        ------------------------------------------- - 3'
Genome                 GCATGTTTCCAGAGAAGACCTGCTGGAGCTGGGGGCTGGGGCA - 3'
                       ***********************************
```

3' End [Exon 3; 3' end with encoded polyA]

```
Leat1 Clone       5' - ATTGTTTACTTCATCAAAAAAAAAAAAAAAAAAAAAAAA---- - 3'
ucsc_ak042353.1   5' - ATTGTTTACTTCATC---------------------------- - 3'
Genome            5' - ATTGTTTACTTCATCATTAAAAAAAAAAAAGAACCCATATTCA - 3'
                       ****************  ************ **    * *
```

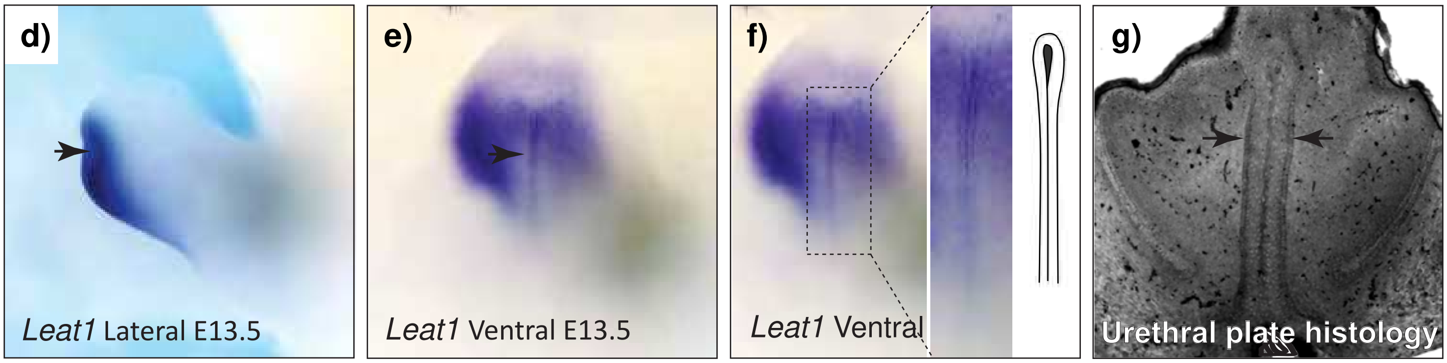

h)

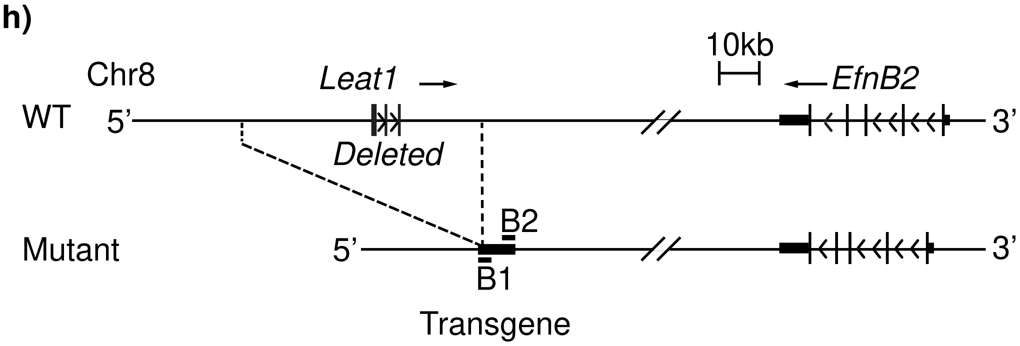

I

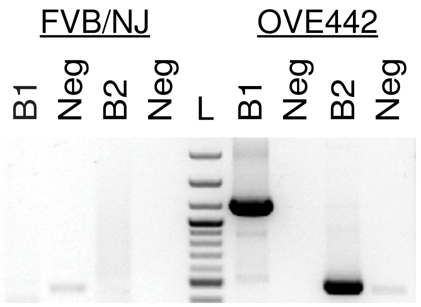

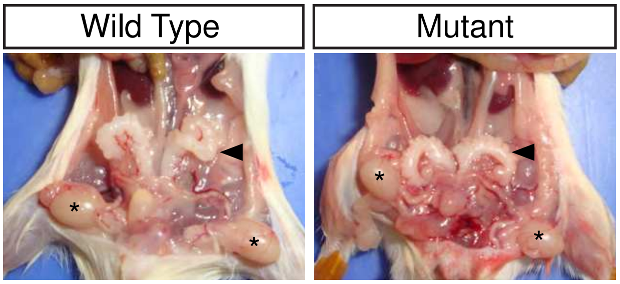
Wild Type
Mutant

a)

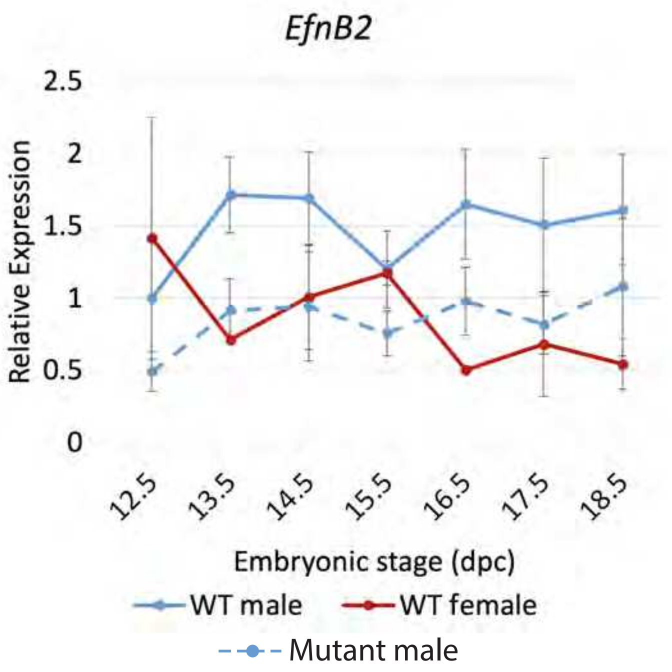

EfnB2
2.5
2
1.5
1
0.5
0
Relative Expression
12.5
13.5
14.5
15.5
16.5
17.5
18.5
Embryonic stage (dpc)
WT male
WT female
Mutant male

b)

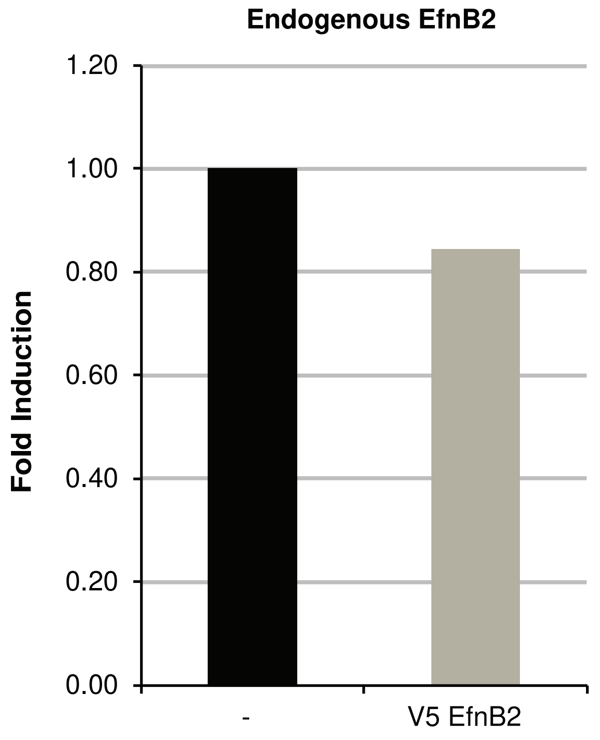

Endogenous EfnB2
1.20
1.00
0.80
0.60
0.40
0.20
0.00
Fold Induction
-
V5 EfnB2

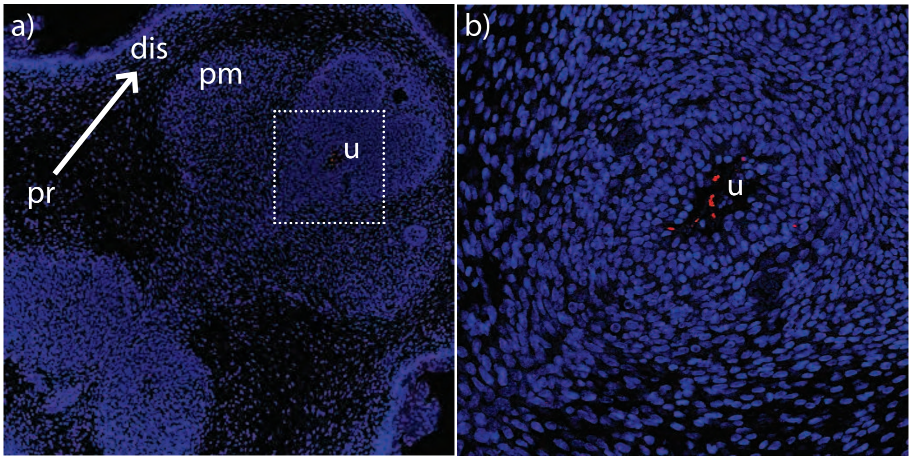

DAPI EfnB2-Leat1

Alignment quality color range: BAD AVG GOOD

Leat1 Alignment Exon 1

```
Mouse    AGACTCAAGCTTTGCCTCCCGCTGGAAAAGGACCTGGGTCACAGTGTCTGAGTCTGTATACGCTGCCACCAA
Human    GCC-------------------------------T--------------------G----------------
Wallaby  GAAT--------------------------------------------------------------------
cons

Mouse    AGGCTGACTTACACAATTGTTCTGATCGCAGTTTTGAACCTGATGTCTGCGCCACCTTACCTGCCGCATACT
Human    -------------------TTC-------A------------------------------------------
Wallaby  ------------------------------------------------------------------------
cons

Mouse    TTGAAGACTAGGGTTGCACCTTCTGCGAGGGCAGAGGAATCCTGTCACTGACAGACCACCTTCGAATCCCTC
Human    ----------G-------------------GCA-----A---------------------------------
Wallaby  ------------------------------------------------------------------------
cons

Mouse    GCTTGGTGAAGTGTGACTTCTAAGTGCTAGACCAGCTTCTAAGAACAGCCTACTTTCTACTTGCAGCTGAAA
Human    -----------T--GAC---------------------------------TACTT-----------------
Wallaby  ------------------------------------------------------------------------
cons

Mouse    ACGAAGGTTGTGTGCTATCGTGAATCGGATATTAGTCGTTAACGAGACGCTCCTTGATAGGAGGTGGCTGTC
Human    -------------------------------TTAGG-------------------------------C----
Wallaby  ------------------------------------------------------------------------
cons

Mouse    CTTGGCCATGTGCTTCCTGAAGCCTTGAAGAAAAGCCACAGCATTTCCTGCTGGTGCCGCTCCTGGGGTGGC
Human    -------A----------G-----------------------------------------------------
Wallaby  ------------------------------------------------------------------------
cons

Mouse    ATCACCAGGCTTTGTTGCACCTCATACTGGATACGTGACCACGGTTGCCAGCTTCTCTCTCCGTGTCATTAT
Human    ------------------------------------------------------------------------
Wallaby  ------------------------------------------------------------------------
cons

Mouse    CATGGAACCTACATGCTGTGACCACTGTGATTTCTGACCGGCTATCACTCAGCCTGCTCAGGCATTGACTAT
Human    ------------------------------------------------------------------------
Wallaby  ------------------------------------------------------------------------
cons

Mouse    TTTTAGGCAGAAGGTCTAGTCCCCGCTTTTATTCCCACAGCCGGCTTCTATTTTAATGCTCCATTTGGCTCC
Human    ----------AGGTTCTAGTCCCTAGTTTTATTCCCAGAGCCGTCTTCTATTTTAATGCTCCATTTTGCTCT
Wallaby  -----------------------------------------------------TAAT---------------
cons                                                          ****
```

```
Mouse     AGGAACCAAACCATAGCCGTGGAATTCCCACAGGGCGTTAACAGAAAGCAAAACCTCCTCCCACTTTGGAAT
Human     AAGAACCGAACCATGCCCATAGAAGTCCCACAGGATTTTGTAGAAAGGCAAACTTCCTCCCACGCCGGAAT
Wallaby   -------GAACCATCC-------------------------------------------------------
cons              ******
```

```
Mouse     TAATGAACCCCTCTCGCTGTCAGCCATGCTTTAATGGATTTCACCAGTCTCTGGATTTCCACACCCAGAACA
Human     TAATGAGCCCCTCTTGCTGTCAACTGTAGTTTAATGGATTTCACCATTCTGTGTATTTTCACACCCACAACA
Wallaby   -------------CTGCTGTCAGCTCTGCTTTAATGGGTTTCACCATTCTGTATATTTTCATACCCAGAACA
cons                     ******* *  *  ******** ******** *** *  **** ** ***** ****
```

```
Mouse     GA-AAGTCATACAAAG
Human     GA-GAGTCATACAAAG
Wallaby   GAAAAGTCATAC--AA
cons      **  ********  *
```

Leat1 Alignment Exon 2

```
Mouse     CCCCCAGCTCCAGC--AGGTCTTCTCTGGAAACATGCCAGTCAAGCCTGCAGGAG-AAGCAATTCAGCCAGTCAA
Human     GTGCATATTTCAAGGGAAGAAGTTTCAAAAAATGAAAAATGAAGGCC-TAGGAGGGTACAGATTATGAAG--GA
cons         *    * **    * *   * **   ***      * * *** *   *****    **  * *   **   *
```

```
Mouse     GCAG
Human     TGAA
cons        *
```

Leat1 Alignment Exon 3

```
Mouse     CAAACACC---ACCA---TGAAAGG---ATGAAGAGA---TAGGATATGCAAAAGGAGG-AGATGTTGGCAGTGA
Human     CAAAGATATGGAACAAGCCCAAATGCCCATCAATCAACAAGTGGATAAAGAAATTGTGGTAAATATATATCATGG
cons      **** *     * **     *** *   ** **   *     *****   ***  * ** * ** *      **
```

```
Mouse     CGTGTTTCTAACTTGTTAACAAGACTGCAGTA--------TCTATCAGCTTCGG-GAGCAGGGCACGAGGTTGGG
Human     AATACTACTCAGCCATAAAAAGGAATTAAATAATGGCATTTGCAGCAGCCTGGAAGTGGAGACCATTACTCTACG
cons        *  * ** *    * ** * ** *  * **        *  * **** * *  * * **  **  *   *  *
```

```
Mouse     AG--------GAAGAATGGT-GGCAAGATTCTGAAAGCTACTGGGCTATTTGT-TGAGATAAA-AATGGGGATGG
Human     TGAAGTAATTCAGGAATGGAAAACCAAACATTGTATGTT-CTCGCTCATAAGTGAGAGCTAAGATATGAGGATGC
cons       *         * ******    * * *   ** * * * ** *   **  **  *** ***   *** *****
```

```
Mouse     GCATCTCCAAACTT--CCCTTTATCCTTCCTTGGATGAAGAAAAATTAA-AGTTAATTTGTGTTCCTTAAAGAAA
Human     AAAG--ACAAGAATGATACAATGGACTTTGGGGAGTCAGGTGAAAGGGTGAG-AGAGGGGTGAGGGATAAAAGAC
cons        *    ***   *    *  *   ***    *  * * *  ***     **   *   ***     ****  *
```

```
Mouse     CACAATTGTTGGGGA-AATGTGGTCCCCTCAC--AAAGGAAAAGCC---ATCTAAGCTAATAGCATCAAT-----
Human     TACA--CGTTGGGTACAGTGTACACTGCTTAGCTGATGGGTCCACCAAAATCTCAGAAATCACCACTAAAGAACT
cons       ***   ****** * * ***   *  ** *    * **     **   **** **  *  * **  **
```

```
Mouse  -TTTCATCACAGTGATT-GG-ATTCTTACCTAAGAA---------ACGGCCCTCTG-GGTTCCTTCAGGGAAGAA
Human  TATTCATGTAACCAAACACCACCTGTTTCCCAAAAACCTATTGAAATTAAAAAATAATAATAATAAAAAGAAAAA
cons     *****   *   *        * ** ** ** **         *        *     *  *  *  *** **

Mouse  TGTTCTT---TCATAAAA--AGACCCA---GGAAAAGAACTTGCCATGAAACCCCAGGATTATCAGCCACACCAT
Human  TATTATTATCTAATAAAAAGAGACCCACAGGGTGAGGAACTTGCCATCCCATCCCAGAAACATCCCTTCCTGTAC
cons   * ** **   * ******  *******   **  * ***********   * ***** *  ***     *   *

Mouse  GTCACAGTGGCTTACT-CTACT---GAC-------TTAC---CTCATGCAAGTGTCACCTGCCACGGTGAGACCA
Human  ATCCCAATGATTTTCAGCCATTAGATACGAATATCAGAGTTTATGATGTTTGCATCAACTGCCATTTTAAGACCA
cons    ** ** **  ** *  * * *    **         *     * ***   *  *** ******   * ******

Mouse  CAGAGGGAAGACTGGCAGGTGGATCTGCACCCTACAGTGATCCATCCTTCAGAAGGATGACTG-----TGGCTTC
Human  ---AGAGAAGACTGAAAGAGAAGTCAGAAACCTGAAGCCATCAA-ACTGAAGATGAATAGCTGGTGAATAGCTGC
cons      ** ********  **     ** * * ***  **  *** *  **  *** * **  ***     * *** *

Mouse  -CTCCTCC---AGTCAGGAAATAGTGTGTGGTCTATTGATCACGCCTCTGCTGTTCAGG-CTCATGACTAGAGGC
Human  CCTTCTCTTTATGTGAAGAAA-AGTAAGCTG-CCGTTGTCTACATTTCAATTAATCAAGTATTATTACTTGTAGT
cons    ** ***     ** * **** ***  *  * *  ***   **   **   *  *** *  * ** *** *  *

Mouse  TGGGAATGCCTGAAGTGATACACAGACATTCTAAACATGAAAGCAGGCATCACACCTGAGAAAATGGCCTGATAC
Human  TAAAAGCAGTTTAACAAATACACAAACCTTCTAGATACAAAAACAAAACTCACCCCTCAGGGGTCAGCTCCAGA-
cons   *   *     * **   ******* ** ***** * *  *** **    **** *** **      **   * *

Mouse  TTCTAAGGGATCAGCAACACTGCTGGTTGCCTTGGTGAGAACAGTGAGGAAACAGGACATCACCTCCTACACTGG
Human  TTCTAGAGGATTTGTCACACAGCTGGTCACCTTGGTGGGACCAGTGAAGAAATGGGATGCAATCTCTTATACCGA
cons   *****  ****  *  **** ******  ******** ** ****** ****  ***    * *** ** ** *

Mouse  AGTCCCTC--CTGCCCCTGTGACTGTCCTAGT-----AAC-TAG-----------CAAGAAGT-----AAAGTGT
Human  AGTTCCTTCACTGCCTCCATCACTGTTCTAAAGAGACAACATAGTAAACTCAAAGCAATACATTCTTAAAAGTAT
cons   *** ***   ***** *  * ***** ***       *** ***           *** *  *     ***** *

Mouse  CG---TATATAAAAC---AAG-ACATGAATTGTAGCAAGATCAAACCCTCCGTGATCGGGTTATGGTTGCATTTC
Human  TATTTCATGTAAATTTATATGTAGATGAATTACAACAAGATGAAACCCTTGGTGGTGGGTTAGTGGTTGCATTTA
cons         ** ****     * * * *******  * ****** *******  *** * ** *  ***********

Mouse  ACAAGA--TTGCTTGACTTCATTTCTAATTTGTTGAATTCCCAGCATATACTACTCTTATAATCAGAAAATAACA
Human  ATTTTCTTTTGCCATTGTCCGTTTTTAATTTATTGAATTTTCAGCGTATGCTACTTTCACAATCAGAAAATGA--
cons   *       ****     * * *** ****** *******  **** *** ***** * * *********** *

Mouse  CAAATATGACTTCTAGAGTGAAAAAAAATGTAGTTCCCACATTATCATTTCAACACTAATCAAGAACCAAAAATA
Human  CAAATATTATCTATAAA----ATAAAAGTATAGCTTCTGCCTTATCATTTCAACACCAATCAAGAACCAAAAGC-
cons   ******* *  * ** *    * **** * *** * *  * *************** ***************

Mouse  AAGGTGGCGCCGGGCAGCAGACCTTAAATGAGAGGCTGTTTTCCCAAGCTCTGGTTAAGATAGGAATGTGAGATA
Human  AAGGTGGCACCAGTCAGCAAGCCTTAAATTAAAGGGTGTTTTCCCAGTTTCTGGTTAAGATAGGAATGCAAGACA
```

```
cons     ******** ** * *****  ******** * *** **********   ******************* *** *

Mouse    CAGCTGAGGTGACATGTGTGAAT-CACCCAGCATGTTCCCAACAACCACAGCTTTAAAGCAACATCCCTCATTCT
Human    CAGTAAAGGTGACAGGTATGTATTTACACAGCACAGTCCCAACAAC-----------------------------
cons     ***   ******** ** ** **  ** *****   **********

Mouse    CTCCCCTGCTTTGTGTAATTCTACATACAAAGCTGTCTTTCTGGCTTCTTCTCTATCCCGTTATCCCTAATAGTT
Human    ----------------------------AA-----T---------------------------------------
cons                                 **     *

Mouse    CCACAGATTCTATATAAGGGCTATTAATACACTAAAAGGACAAAATTGTTTACTTCATCA
Human    -CATAG--------------CT-T--------T-AAAG-----------------C--AA
cons      ** **              ** *        * ****                 *   *
```

Supplementary Table 1

mLeat1 cloning primers	Clm353F, Forward 5'- GTACTAGCTAGCAAGCTTTGCCTCCCGCTGGAAAAGG -3'
	Clm353R, Reverse 5'- ATAAGAATGCGGCCGCGATGAAGTAAACAATTTTGTCC -3'
mEfnB2 CDS cloning primers	ClmEfnB2F, Forward 5'- CACCATGGCCATGGCCCGGTC -3'
	ClmEfnB2, Reverse 5'- GACCTTGTAGTAAATGTTGGCAGGACTC -3'
mEfnB2 in situ probe	ClimEfnB2F, Forward 5'- CAAATGGGTCTTTGGAGGGC -3'
	ClimEfnB2R, Reverse 5'- CCCAGGGTCGGAAAAGCTAC -3'
Genotyping OVE primers mutant	OVEMutFwd, Forward 5'- CCACATTTGTAGAGGTTTTACTT -3'
	OVEMutRev, Reverse 5'- CCCTGGTATCACTGAACAATCA -3'
Genotyping OVE primers wild type	OVEWTFwd, Forward 5'- GACATGCCTGTTCATATCTTGGC -3'
	OVEWTRev, Reverse 5'- CTTTGAGGCTCTTTGCATTGTGA -3'
Transgene insertion Genomic boundary 5'	B1F, Forward 5'- AGGAATTGGGGTTAATGGTTGGT -3'
	B1R, Reverse 5'- AGCCATACCACATTTGTAGAGGTT -3'
Transgene insertion Genomic boundary 3'	B2F, Forward 5'- CCACATTTGTAGAGGTTTTACTTGC -3'
	B2R, Reverse 5'- CCCTGGTATCACTGAACAATCA -3'
Leat1 RNA orientation RT-PCR	OuterRT353F1, Forward 5'- TGCTCAGGCATTGACTATTT -3'
	InnerRT353R1, Reverse 5'- ATGCCCATCCCCATTTTTAT -3'
	InnerRT353 F2, Forward 5'- GTCTAGTCCCCGCTTTTATT -3'
	OuterRT353 R2, Reverse 5'- TCCAAGGAAGGATAAAGGGA -3'
	V5EfnB2, Forward 5'- CAAGGTCAAGGGCAATTCTGCAGATATCCAG -3'

Supplementary Table 1

Exogenous EfnB2 qRT-PCR	V5EfnB2, Reverse 5'- TCAATGGTGATGGTGATGATGACCGGTACG -3'
Endogenous EfnB2 qRT-PCR	EndEfnB2, Forward 5'- CAAGGGAACTCGCACCTTGTTCTTGGGCAC -3'
	EndEfnB2, Reverse 5'- GTGGGGATCTCCTAGCAGTCTTCCAGCTTC -3'
Actin qRT-PCR	Actin, Forward 5'- CAACTGGGACGACATGG -3'
	Actin, Reverse 5'- GCAACATAGCACAGCTTCTC -3'
Hprt qRT-PCR	Hprt, Forward 5'- GAGGGTCCTGTTGATGTGCCAG -3'
	Hprt, Reverse 5'- GGTGGCTTAGGCTCATAGTGC -3'
Leat1 qRT-PCR	Leat1, Forward 5'- TCGCAGTTTTGAACCTGATG -3'
	Leat1, Reverse 5'- GCTGGGCTTTTCTTCAAGG -3'
EfnB2 qRT-PCR	EfnB2, Forward 5'- CTCAACTGTGCCAGACCAGA -3'
	EfnB2, Reverse 5'- GGATCCAGGCCCTCCAAAG -3'
Arid1B qRT-PCR	Arid1b, Forward 5'- GCCGCGCAACAAAGGAGTC -3'
	Arid1b, Reverse 5'- AGTAGCCACTCACAGCTTGC-3'
Cux1 qRT-PCR	Cux,1 Forward 5'- GCTGAACACCCTGAAGTCCA -3'
	Cux1, Reverse 5'- CCCTTTTCTCCTGGCTGACC -3'
Frmd4 qRT-PCR	Frmd4, Forward 5'- AGCTCTTCTTCCTGAATGCCAA-3'
	Frmd4, Reverse 5'- GTAGCGCTGGCAGCTTTTTC-3'
Aust2 qRT-PCR	Aust2, Forward 5'- TCACCCCAATATGTTCGCCC-3'
	Aust2, Reverse 5'- CTCTGAGAGGCCAGGAAACG-3'

Supplementary Table 1

Frem2 qRT-PCR	Frem2, Forward 5’- GAATTGGGCGTGGATCTCCTT-3’
	Frem2, Reverse 5’- AGGCTCCGAGAGAACCACTT-3’
Ryr2 qRT-PCR	Ryr2, Forward 5’- AGCTGGAAGACCCTGCAATC-3’
	Ryr2, Reverse 5’- ACCAGGCTGAAATATCCCCG-3’
Sim2 qRT-PCR	Sim2, Forward 5’- TTCCCGGAAGGTCTAGGAGA-3’
	Sim2, Reverse 5’- GCCACCACGAACACAAATCC-3’
Mafb qRT-PCR	Mafb, Forward 5’- TTCGACGTGAAGAAGGAGCC-3’
	Mafb, Reverse 5’- GTAGTTGCTCGCCATCCAGT-3’
